# Association of particles of lipoprotein subclasses with arterial stiffness in a high-risk working population: the Baptist Employee Healthy Heart Study (BEHHS)

**DOI:** 10.1186/s43044-020-00046-4

**Published:** 2020-03-19

**Authors:** Muhammad Aziz, Swetha Gannarapu, Choudhry Humayun, Usman Siddiqui, Khurram Nasir, Ehimen C. Aneni

**Affiliations:** 1 Family Medicine Center, West Kendall Baptist Hospital, Miami, FL USA; 2 Center for Healthcare Advancement & Outcomes, Baptist Health South Florida, 9555 SW 162nd Ave. MAB, Suite 103, Miami, FL 33196 USA; 3 Florida International University, Department of Humanities, Health, and Society (HHS), Herbert Wertheim College of Medicine, Miami, FL USA; 4 Center for Outcomes Research and Evaluation, Yale-New Haven Hospital, New Haven, CT 06510 USA; 5 Johns Hopkins University, Johns Hopkins Ciccarone Center for Prevention of Heart Disease, Baltimore, MD USA; 6 Internal Medicine, Section of Cardiovascular Medicine, Yale-New Haven Hospital, New Haven, CT 06510 USA

**Keywords:** Lipoprotein, Lipoprotein subclasses, Arterial stiffness, Augmentation index, Employee population

## Abstract

**Background:**

Arterial stiffness is an independent predictor of cardiovascular disease (CVD) morbidity and mortality. A risk factor-independent association of arterial stiffness with traditional lipids has been described extensively, but it is still unclear whether an independent relationship exists between arterial stiffness and particles of lipoprotein subclasses.

**Methods:**

The Baptist Employee Healthy Heart Study (BEHHS) is a lifestyle intervention study examining the effects of web-based programs on reducing CVD risk in high-risk persons. Participants had their brachial arterial augmentation index (AI, a measure arterial stiffness) assessed using the EndoPAT 2000 device. Cardio IQ™ ion mobility lipoprotein fractionation was utilized for measurement of particles of lipoprotein subclasses.

**Results:**

The population consisted of 182 participants, (74% women, 49% Hispanic) with a mean age of 52 ± 9 years. There was a significant trend association between quartiles of AI and total cholesterol, HDL-c, large LDL-p, small IDL-p, large IDL-p, and all subclasses of HDL particles (total HDL-p, small HDL-p, and large HDL-p). In logistic regression analysis, only HDL-c, total LDL-p, large LDL-p, small IDL-p, large IDL-p, total HDL-p, small HDL-p, and large HDL-p demonstrated significant independent association with AI.

**Conclusion:**

Several lipoprotein subclasses demonstrate independent significant associations with arterial stiffness. A safe and relatively inexpensive blood test may be useful in identifying subclinical atherosclerosis process in a relatively young high CVD risk population.

**Trial registration:**

ClinicalTrials.gov, NCT01912209. Registered July 31, 2013

## Research highlights

- Cardio IQ™ ion mobility lipoprotein fractionation is a better estimate of particles of lipid subclasses as compared to traditional lipid measurement.
- This study is a step toward determining the true relationship between advanced lipid panel (particles of lipoprotein subclasses) and augmentation index, a measure of arterial stiffness.
- Several lipoprotein subclasses demonstrated independent significant associations with arterial stiffness.

## Background

Arterial stiffness, an age-related irreversible hardening of vessels, is associated with CVD-related morbidity and mortality [1–4]. Arterial stiffness can be measured by augmentation index (AI) and pulse wave velocity (PWV) that represent as measure of wave reflection and arterial stiffness [5, 6]. AI increases with age and is closely associated with CVD risk factors such as hypertension and coronary artery disease [7, 8]. It also reflects properties of cardiac performance and overall ventricular-vascular coupling [4, 9, 10]. Brachial artery AI is a measure of both central and peripheral arterial stiffness [6, 11].

The association of arterial stiffness with traditional lipids, such as total cholesterol (TC), triglycerides (TG), low-density lipoprotein cholesterol (LDL-c), and high-density lipoprotein cholesterol (HDL-c), has been described in cross-sectional studies among high-risk population [12, 13]. However, the relationship between arterial stiffness and particles of lipoprotein subclasses has been rarely studied. The aim of this study is to determine the relationship between traditional lipids and lipoprotein subclasses (measured by the ion mobility technique) with brachial artery AI, a measure of arterial stiffness, in a relatively young high-risk employee population.

## Methods

The Baptist Employee Healthy Heart Study (BEHHS) is a randomized controlled trial examining the efficacy of an interactive internet-delivered program on reducing metabolic risk in an employee population with diabetes and/or metabolic syndrome (MS). The design and selection criteria for BEHHS have been described in detail previously [14]. The results of this analysis are based on the data from baseline visit of the study. The study protocol was approved by the Baptist Health South Florida Institutional Review Board (IRB#13-028). All participants have provided written consent to participate in the study.

The study measured lipoproteins using the ion mobility technique, a gas chromatography technique that was adapted to measuring subclasses of lipoproteins and has been validated in several studies [15, 16]. Details of the technique have also been previously described [17]. Augmentation index as derived from peripheral arterial tonometry (endoPAT Itamar Medical Ltd., Israel) is calculated from reflected pulse wave indices. It has shown good test-retest reliability in subjects with metabolic syndrome in previous studies [18]. Because the AI is dependent on the heart rate, it is normalized to a heart rate of 75. The values provided are gender matched [19].

All statistical analyses were performed using STATA version 13 statistical analysis package (StataCorp LP, College Station, TX) [20]. Continuous variables were expressed as means ± SD or median (interquartile range). Categorical variables were represented by frequency and percentage. Spearman Rank correlation coefficients were calculated for each class of lipids with AI (continuous variable). The logistic regression model (controlling for age and BMI) was used to compute the odds ratios and 95% confidence interval (CI), for an increase in AI with each standard deviation increase in lipoprotein particle.

## Results

The mean age of the 182 study participants was 52 ± 9 years with 74% women (n = 134) and 49% Hispanic population. Across increasing quartiles of AI, there was a significant trend with increasing age and a greater proportion of females. By contrast, BMIs decreased with higher AI (Table 1). HDL-c was the only traditional lipid correlated with AI. Among the lipoprotein subclasses, Spearman correlation showed that small IDL-p, total HDL-p, small HDL-p, and large HDL-p were significantly associated with AI (Table 2). Total cholesterol, large LDL-p, and large IDL-p were also significantly correlated with AI. Logistic regression analysis showed that increases in total LDL-p, large LDL-p, small IDL-p, large IDL-p, total HDL-p, small HDL-p, and large HDL-p were significantly associated with increase in AI in the fully adjusted model.

**Table 1:** Descriptive characteristics of the study population along the quartiles of augmentation index (Al).

Variables [mean (SD) or %]	All (*n* = 182)	Q1	Q2	Q3	Q4	*P* trend
Age in years (SD)	51 (10)	46.26 (9.6)	49.42 (9.18)	52.97 (9.96)	55.91 (7.83)	**< 0.0001**
% female	74	41.30	77.78	82.22	93.33	**< 0.0001**
Race						
White	77.1	68.89	93.18	78.57	68.18	0.503
African American	16.6	26.67	4.55	11.90	22.73	
Other	6.3	4.44	2.27	7.14	9.09	
BMI [kg/m2] (SD)	34.2 (6)	37.20 (6.3)	35.20 (5.7)	33.11 (5.3)	31.26 (5.1)	**< 0.0001**
SBP [mmHg] (SD)	134 (15.2)	132.52 (12.1)	133.38 (13.6)	134.64 (16.5)	137.09 (17.5)	0.343
DBP [mmHg] (SD)	83 (9.2)	84.06 (8.5)	82.53 (8.5)	82.36 (9.6)	82.02 (10.2)	0.288
Heart rate [HR]	73 (11)	72.39 (11.0)	74.58 (9.1)	74.38 (12.7)	70.76 (11.3)	0.278
Glucose [mg/dL]	105 (37.3)	99.20 (27.5)	106.53 (46.5)	114 (45.7)	100.17 (23.5)	0.325
CRP [mg/L]	6 (7.8)	7.24 (12.8)	5.81 (5.7)	6.26 (6.1)	4.08 (3.0)	0.355
% diabetes mellitus	29	21.74	37.78	31.11	24.44	0.950
% lifetime smoker	34	26.67	31.11	44.19	35.56	0.215
Physical activity [min/ day]	1.68 (0.5)	1.62 (0.5)	1.67 (0.5)	1.68 (0.5)	1.73 (0.5)	0.303

Values are expressed as means ± S.D. or percentages. *BMI* body mass index, *SBP* systolic blood pressure, *DBP* diastolic blood pressure, *Hs-CRP* high-sensitivity C-reactive protein

**Table 2:** Association of lipoprotein (LP) subclasses with augmentation index (Al) quartiles in study population (expressed as IQR)

	AI continuous	AI in ascending quartiles	
	Spearman correlation	OR per SD increase in lipid/lipoprotein	
		Unadjusted	Adjusted
TC [mg/dL]	0.1639*	1.30 (0.99–1.71)	1.21 (0.91–1.60)
TG-c [mg/dL]	0.0000	.99 (0.77–1.27)	0.97 (0.74–1.26)
HDL-c [mg/dL]	0.2298**	**1.56 (1.19–2.04)**	1.19 (0.89–1.60)
LDL-c [mg/dL]	0.0524	1.07 (0.82–1.40)	1.11 (0.84–1.46)
Non-HDL-c [mg/dL]	0.0755	1.18 (0.91–1.54)	1.23 (0.93–1.61)
Total LDL-p [nmol/L]	0.1093	**1.31 (1.0–1.73)**	**1.42 (1.07–1.89)**
Very Small LDL-p [nmol/L]	0.0150	1.18 (0.90–1.54)	1.27 (0.97–1.67)
Small LDL-p [nmol/L]	0.0133	1.12 (0.86–1.47)	1.26 (0.95–1.66)
Medium LDL-p [nmol/L]	0.0605	1.23 (0.93–1.61)	1.37 (1.03–1.82)
Large LDL-p [nmol/L]	0.1837*	**1.44 (1.10–1.89)**	**1.40 (1.05–1.86)**
Small IDL-p [nmol/L]	0.2643**	**1.50 (1.13–1.98)**	**1.40 (1.05–1.87)**
Large IDL-p [nmol/L]	0.1707*	**1.41 (1.05–1.88)**	**1.49 (1.11–2.00)**
Total HDL-p [nmol/L]	0.2747**	**1.48 (1.10–1.99)**	**1.41 (1.03–1.94)**
Small HDL-p [nmol/L]	0.2507**	**1.45 (1.09–1.93)**	**1.40 (1.03–1.90)**
Large HDL-p [nmol/L]	0.2658**	**1.48 (1.10–2.00)**	**1.37 (1.00–1.89)**
Small VLDL-p [nmol/L]	0.1203	1.32 (0.99–1.77)	1.43 (1.06–1.94)
Medium VLDL-p [nmol/L]	0.0451	1.19 (0.90–1.57)	1.30 (0.97–1.74)
Large VLDL-p [nmol/L]	− 0.0052	1.13 (0.85–1.50)	1.16 (0.87–1.55)

Adjusted for age and BMI. Spearman *ρ* = Spearman rank correlation coefficient using Bonferroni significance level. Cut-off *p* value of *p* < 0.05 was used for determining significance. Logistic regression models using 95% CI describe the change in AI per SD change in standardized values of lipoproteins. *AI* augmentation index, *LP* lipoprotein, *p* particle number, *TC* total cholesterol, *TG* triglycerides, *LDL* low-density lipoprotein, *IDL* intermediate-density lipoprotein, *HDL* high-density lipoprotein, *VLDL* very low-density lipoprotein

*p value ≤ 0.05

**p value ≤ 0.005

## Discussion

Our study findings, that associate higher LDL particles (total and large) with AI, differ slightly from earlier studies which showed a significant positive association of baPWV (a measure of arterial stiffness) with lipoprotein subclasses of total LDL-p and small LDL-p in relatively healthy population (age 40–49 years) [21] and with very small LDL-p in patients with impaired glucose metabolism [22]. The reason for these varied results among these studies could be due to use of different methods to examine the peripheral arterial stiffness and use of different laboratory techniques to measure the subclasses of lipoproteins.

HDL particles do not follow traditional inverse relationship with CV risk factors, therefore complicating proper assessment of the relative roles of distinct HDL particles in cardio-protection [15, 23, 24]. We discovered that all subclasses of HDL showed a significant positive association with AI in adjusted models. This finding was not unique to our study as previous published papers describe a positive relationship of HDL particles with AI [21, 25, 26].

Neither triglycerides nor the triglyceride-rich VLDL particles were associated with the AI in our study population. The reasons for this unexpected lack of relationship are not readily clear. Previous studies had shown strong associations of VLDL particles with peripheral arterial stiffness [21]. Our study showed a paradoxical trend toward decreasing BMI with increasing AI. Our study is not the first to report this negative association of obesity with augmentation index [27]. For this reason, we controlled for BMI in the lipoprotein statistical analyses.

Our study is limited by the small sample size which diminishes the study’s power. The cross-sectional nature of the study precludes conclusions on causality. The largely Hispanic population also limits the generalizability of the study’s findings. We only measured the association of lipids with measures of peripheral arterial stiffness. The inherent variability of the AI as a measure of overall vascular function may indicate that comparison of different methods of AI is needed to predict the association with all classes of lipids.

## Conclusions

In conclusion, our study shows that measures of lipoprotein subclasses are associated with increased peripheral arterial stiffness as measured by AI in a high metabolic risk population. Our study represents a step toward determining the true relationship between lipids and arterial stiffness. Once this relationship is clarified further, pharmacological and lifestyle interventions can be studied in the context of their effect on levels of these lipoprotein subclasses, with the aim of correcting these abnormalities in time and potentially preventing further CVD events.

## Data Availability

The datasets used and/or analyzed during the current study are available from the corresponding author on reasonable request.
